# Population movement can sustain STI prevalence in remote Australian indigenous communities

**DOI:** 10.1186/1471-2334-13-188

**Published:** 2013-04-25

**Authors:** Ben B Hui, Richard T Gray, David P Wilson, James S Ward, Anthony M A Smith, David J Philip, Matthew G Law, Jane S Hocking, David G Regan

**Affiliations:** 1 The Kirby Institute, University of New South Wales, Sydney, NSW 2052, Australia; 2 Baker IDI Heart and Diabetes Institute, Alice Springs, NT 0871, Australia; 3 Australian Research Centre in Sex, Health and Society, La Trobe University, Melbourne, Victoria 3000, Australia; 4 Centre for Women’s Health, Gender and Society, The University of Melbourne, Carlton, Victoria 3053, Australia

**Keywords:** Mobility, Indigenous population, Remote communities, Chlamydia, Gonorrhoea

## Abstract

**Background:**

For almost two decades, chlamydia and gonorrhoea diagnosis rates in remote Indigenous communities have been up to 30 times higher than for non-Indigenous Australians. The high levels of population movement known to occur between remote communities may contribute to these high rates.

**Methods:**

We developed an individual-based computer simulation model to study the relationship between population movement and the persistence of gonorrhoea and chlamydia transmission within hypothetical remote communities.

**Results:**

Results from our model suggest that short-term population movement can facilitate gonorrhoea and chlamydia persistence in small populations. By fixing the number of short-term travellers in accordance with census data, we found that these STIs can persist if at least 20% of individuals in the population seek additional partners while away from home and if the time away from home is less than 21 days. Periodic variations in travel patterns can contribute to increased sustainable levels of infection. Expanding existing STI testing and treatment programs to cater for short-term travellers is shown to be ineffective due to their short duration of stay. Testing and treatment strategies tailored to movement patterns, such as encouraging travellers to seek testing and treatment upon return from travel, will likely be more effective.

**Conclusion:**

High population mobility is likely to contribute to the high levels of STIs observed in remote Indigenous communities of Australia. More detailed data on mobility patterns and sexual behaviour of travellers will be invaluable for designing and assessing STI control programs in highly mobile communities.

## Background

STI diagnosis rates are considerably higher among Indigenous Australians, particularly among those living in rural and remote areas, than in the general population. In 2011, the diagnosis rates for gonorrhoea and chlamydia were 30 times and 3.5 times higher, respectively, than among non-Indigenous Australians [1]. A baseline prevalence study conducted in 2010 reported prevalences for both gonorrhoea and chlamydia of more than 7% for the 16–34 age groups [2]. These levels of STI prevalence are similar to those that have been reported for many remote communities during the 1996–2006 period [3] despite intensive STI screening programs (at least 44% coverage per year and 75% treatment rate) during this period [4]. The persistence of endemic levels of infection in remote communities [2] has been recognised as a public health failure in one of the world’s richest countries and has led to questions about the validity of current approaches for control and prevention [5].

The Indigenous population in Australia is generally much younger than the non-Indigenous population, and is associated with socio economic disadvantage and poorer health service accessibility. These factors likely contribute to high STI diagnosis rates [6]. We hypothesise that an additional factor could be the level of population movement between remote areas, which is known to be high. Using census data, Biddle and Prout [7] determined that the short-term movement, which we will refer to as “temporary mobility”, of Indigenous people ranges from less than 2 weeks to as long as 6 months [8,9]. Mobility rates for most communities peak in 17–25 year-olds who are the most sexually active and have the highest diagnosis rates for chlamydia and gonorrhoea.

We use a model of chlamydia and gonorrhoea transmission in hypothetical populations that have some of the key characteristics of remote Indigenous communities, to explore the possible impact of movement between communities on STI transmission.

## Methods

### General

We developed an individual-based mathematical model of STI transmission to describe a sexually active population aged from 15 to 45 years. The model tracks age, gender, home and current location, infection status, and sexual behaviour on a daily basis. A summary of the modelling methodology follows, with the accompanying Additional file 1 providing full implementation details.

**Figure 1:**
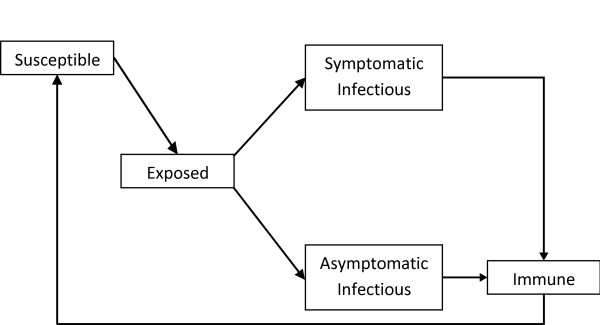
Infection pathway for gonorrhoea and chlamydia as implemented in the model.

An individual’s partner seeking frequency and permitted partnership types are based on the results from a recent study on the sexual behaviour of young Aboriginal people [10]. Table 1 lists the parameter values used. In our model the acquisition and progression of infection for gonorrhoea and chlamydia follow identical pathways — as shown in Figure 1 — and the two infections are distinguished by the appropriate choice of parameter values as summarised in Table 2. Prevalence at time t = 0 was set to approximate the results from a recent baseline prevalence study in remote Australia [2], with the time of onset of each infection determined by random draw from an exponential distribution of infection duration (described in Table 2). The distribution of individuals across the other compartments (disease states – see Figure 1) was determined by the relative mean duration of time spent in each state.

**Table 1:** **Sexual behaviour characteristics of individuals comprising the model population, based on results from Bryant *et al.***[10]**, unless otherwise specified**

**Sexual behaviour parameters**	**Value**
Proportion of individuals that can only form regular partnerships	0.365
Proportion of individuals allowed to form casual partnerships who can have casual and regular partner at the same time	0.276
Proportion of individuals that seek more than 1 partner in 6 months	0.4
Length of average regular partnership	2 years
Probability of condom use per act (neither partner have symptoms)	
Regular partnerships	0.206
Casual partnerships	0.392
Probability of condom use per act (when at least one partner has symptoms)	
Regular partnerships	0.567
Casual partnerships	0.801
Frequency of sex (number of sex acts per week)	3 (assumption based on data for the general Australian population [11])

**Table 2:** Model parameter values and distributions

**Infection parameters**	**Distributions**			
	**Gonorrhoea**	**Reference**	**Chlamydia**	**Reference**
Probability of infection being symptomatic				
Male	β (0.9, 0.05)	[12-15]	β (0.30, 0.15)	[12,13]
Female	β (0.4, 0.15)	[12-15]	β (0.15, 0.08)	[12,13]
Duration of latent period (days)	U (3, 5)	[13,16-18]	U (12, 28)	[13,17,19]
Duration of asymptomatic infection in the absence of treatment (days)	Γ (105, 35)	[12,13,20]	Γ (220, 188)	[12,19,21-24]*
Duration of symptomatic infection in the absence of treatment (days)	Γ (105, 35)	[12,13,20]	Γ (112, 35)	[12,25]
Duration of immunity following recovery (days)	Γ (7, 3.5)	-	Γ (45, 15)	-
Transmission probability per sex act				
Male to Female	β (0.2, 0.05)	[12,21,26,27]	β (0.16, 0.10)	[12,19,28,29]
Female to Male	β (0.4, 0.10)	[12,21,26,30,31]	β (0.12, 0.06)	[12,19,28,29]

Entries with no references are assumptions. U (*l, u*) denotes a uniform distribution with lower limit of *l* and upper limit of *u*; β (*m*, *σ*) denotes a beta distribution with mean of *m* and standard derivation of *σ*; Γ (*m*, *σ*) denotes a gamma distribution with mean of *m* and standard derivation of *σ*. Entries marked with ‘*’ indicate that the parameter has been adjusted in the model calibration process in order to generate realistic prevalences for gonorrhoea and chlamydia.

### Population demographics

We refer to an individual’s usual place of residence as their “home location”. Individuals currently located at their home location are termed residents, and individuals located away from their home location are termed non-residents.

The modelled population consists of 5000 individuals, corresponding approximately to the number of 15 to 45 year old Indigenous people in regions close to towns such as Alice Springs or Tennant Creek in the Northern Territory of Australia [32]. Each individual is a resident of one of five home locations: one location is the home location of 4000 individuals, representing the population of a large regional centre; each of the remaining locations is the home location of 250 individuals representing smaller remote satellite communities [33].

### Population movement

Individuals within the population cannot change their designated home locations, but are able to move to any other location temporarily before returning home. An individual’s destination was determined via a random process, weighted by the population size of each possible destination but not the physical distance between locations. We based this process on findings from studies of Australian remote Indigenous communities showing that mobility is more closely associated with kinship or family ties than with proximity [9,34,35].

The Additional file 1, under the section entitled “Individual movement”, describes the methods used to implement mobility in detail. In summary, the model uses three parameters to describe mobility — *A*_*s*_*, A*_*p*_ and *A*_*d*_ and (defined in Table 3) — which were fitted during model calibration of the proportion of non-residents in the Indigenous population against census data (Table 4).

**Table 3:** **Description and baseline value for parameters *A***_***s***_***, A***_***p ***_**and *A***_***d***_

**Parameter**	**Definition**	**Baseline value**
*A*_*s*_	Probability (per day) of an individual with an existing partnership in their home location seeking a new partner while away from home	0.2
*A*_*p*_	Proportion of the population that contributes to movement	100%
*A*_*d*_	Number of days, on average, an individual stays away from home	14-21

**Table 4:** **Percentage of the population that is non-resident at a given time, by gender and age group, as specified in the model and based on census data for remote communities in Australia**[7]

**Percentage of the population that is non-resident (%)**		
**Age range (Years)**	**Male**	**Female**
15 - 20	10.0	11.1
21 - 25	9.4	10.1
26 - 30	9.5	8.4
31 - 35	9.2	7.9
36 - 40	8.5	8.7
41 - 45	8.5	8.2

When individuals within sexual partnerships move from their home location and are seeking partners, their partner seeking preferences are either the same as in their home location or, with probability *A*_*s*_ they behave as if they are single, and are therefore able to seek new regular or casual partners at their new location. To examine the impact of *A*_*s*_ on STI prevalence, we assumed all other factors relating to an individual’s sexual behaviour — such as the likelihood of forming a casual or concurrent partnership and the frequency of seeking partners — are unaffected by the level of mobility. In other words, assuming a suitable partner can be found, an individual is just as likely to establish a regular or casual partnership as they are in their home location. This assumption is enforced due to a lack of data relating to changes in sexual behaviour of mobile individuals.

While census data enabled us to estimate the proportion of the population non-resident at a given time by age and gender (see Table 4 and [7]), we could not determine if all or only a small proportion of the population contributes to temporary mobility. Therefore, we varied this proportion, measured by *A*_*p*_, to determine its potential impact. Note that all individuals in the population can move when *A*_*p*_ = 1. The overall proportion of the population that is non-resident at a given time is fixed in this model. Therefore, assuming that the average duration of non-residence, *A*_*d*_*,* remains constant, a smaller *A*_*p*_ means that a smaller proportion of the population can contribute to movement, but individuals will tend to move more frequently. For example, if we assume there are 100 individuals that can move in the population, and other parameters are set such that one individual is moving away from home each day, then on average, each individual can move away from home every 100 days. If the number of individuals that can move is doubled, then the average frequency at which individuals move away from home will be halved.

To capture temporary mobility, all non-residents return to their home location after a short period, specified by a predetermined range *A*_*d*_. The upper bound for *A*_*d*_ in our investigation was 180 days because the criteria for residency in census data is at least 6 months and there is evidence of frequent short term movement, lasting from two days to several months, in Indigenous populations [8,9].

While there are currently insufficient data to describe the temporal variations in the proportion of the population travelling at a given time, evidence does exist to suggest that temporal variations in mobility may occur due to changes in road accessibility between wet and dry seasons and the timing of annual cultural events [9]. We investigated the effect of periodic mobility on STI prevalence by varying *A*_*p*_ over a half-yearly cycle.

### STI treatment

In this model, we assumed that the presence of symptoms can influence condom usage as has been shown to be the case for other sexually transmissible infections such as genital herpes [36]. We assume that condoms are widely available in remote communities [10], but access to local health services is more limited [37] such that symptomatically infected individuals are more likely to use condoms than seek treatment to prevent further transmission.

Individuals with symptomatic infection can however seek treatment. This is particularly important for gonorrhoea as a relatively large proportion of male infections are symptomatic in comparison to chlamydia [16,38]. We assume 44% of the population are screened and treated annually, based on a recent review of STI programs in remote Aboriginal communities in Australia [4], and define symptomatic treatment coverage as the percentage of symptomatic individuals treated within 25 days of the appearance of symptoms. There is evidence to suggest that the duration of STI infection is longer for residents of remote Indigenous communities due to limited access to treatment [6]. In this study, therefore, we assume that individuals with symptomatic infection will rarely seek treatment unless a symptomatic treatment program is in place. To provide a reference point for comparison, symptomatic treatment is assumed to be zero in the baseline model and for other simulations where the impact of symptomatic treatment is not investigated. We examined the impact on STI prevalence of different levels of symptomatic treatment for resident and non-resident individuals. We also investigated strategies whereby individuals are encouraged to seek testing and treatment on return to their home location.

## Results

**Figure 2:**
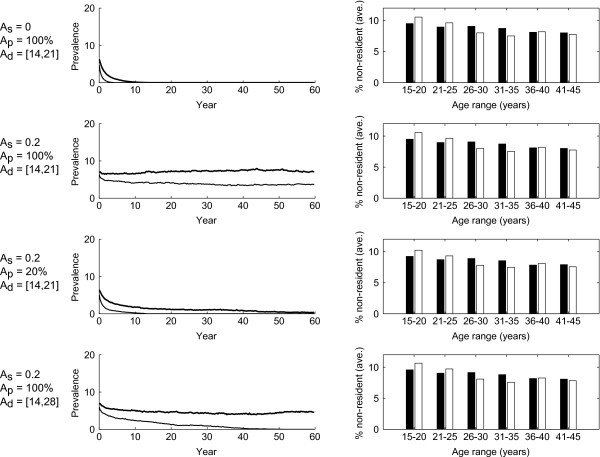
**Representative result from simulations runs.** Each row consists of the result from 100 simulation runs under the same parameter settings and initial conditions. Left column: The median prevalence of gonorrhoea (fine lines) and chlamydia (solid lines) over 60 years. Right column: The average percentage of population away from home over the 60 year period, (males black and females white columns) and age range. First row: Individuals could stay away from home from 14 to 21 days per travel session, but partner seeking behaviour will not change while they are away. Second row: Same as first row, but there is 20% chance an individual who has a regular partner and cannot form concurrent partnership will seek a new partner while away from home. Third row: Same as second row, but only 20% of the population were allowed to move away from home. Fourth row: Same as second row, but individuals could stay away from home from 14 to 28 days per travel session.

Figure 2 shows that, in general, variations in mobility parameters can yield large differences in the prevalence and persistence of STIs, even when the proportion of the population that is non-resident (as shown on the right column) remains largely the same. When *A*_*p*_ and *A*_*d*_ are set to their baseline values with *A*_*s*_*=* 0, (first row Figure 2) gonorrhoea and chlamydia cannot persist at endemic levels beyond 15 years. If all three mobility parameters are set to their baseline values, both STIs persist for up to 60 years (second row Figure 2). The third and fourth rows of Figure 2 show the cases where *A*_*p*_ and *A*_*d*_ are varied from their baseline values, leading to gonorrhoea becoming extinct while chlamydia persists for at least 60 years.

The results that follow examine in more detail the impact of varying the three mobility parameters, as well as the impact of periodic variations in mobility and different treatment strategies, on gonorrhoea and chlamydia prevalence and persistence. All results were obtained from 100 simulations (increasing the number of simulations used did not change the means and 25th-75th percentiles of our results – data not shown).

### Impact of varying *A*_*s*_, *A*_*p*_ and *A*_*d*_

**Figure 3:**
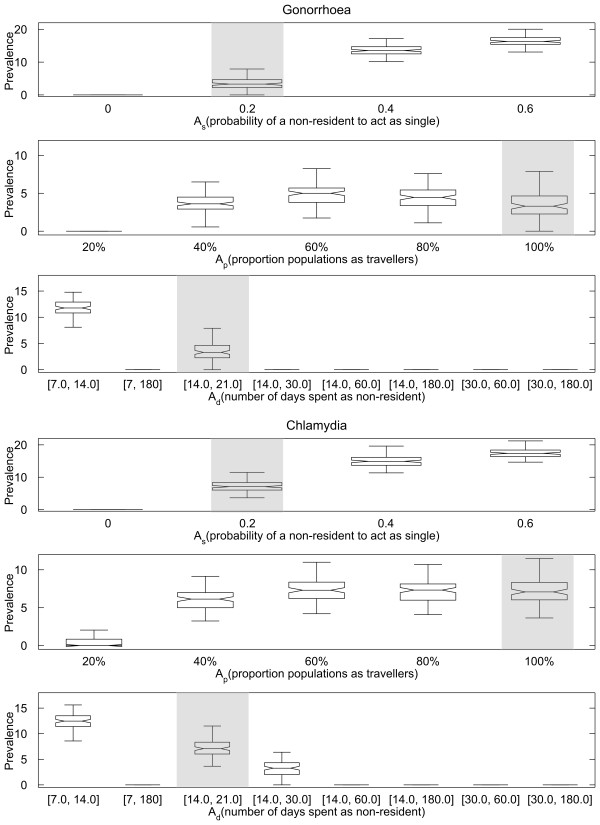
**The prevalence of gonorrhoea and chlamydia at 60 years after introduction of STI under different *A***_***s***_**(with *A***_***p***_**and *A***_***d***_**at their baseline values, first and fourth rows), *A***_***p***_**(with *A***_***s***_**and *A***_***d***_**at their baseline values, second and fifth row) and *A***_***d***_**range (with *A***_***s***_**and *A***_***p***_**at their baseline values, third and sixth row).** Each box represents the 25th-75th percentiles for 100 simulations. The centre line in each box indicates the median. The maximum whisker length is 1.5 times the width of interquartile range (corresponds to approximately 99.3% coverage if prevalence at steady state is normally distributed), and outliers are not plotted. The notch in each box represents the comparison intervals such that two medians are significantly different at the 5% significance level if their intervals do not overlap (see McGill R, Tukey JW, Larsen WA: Variations of Box Plots. *The American Statistician* 1978, 32:12–16). The baseline scenario is highlighted in shade for all rows.

Increasing *A*_*s*_ leads to a significant increase in the median STI prevalence at time t = 60 years (rows 1 and 4 in Figure 3). For *A*_*s*_ = 0 (i.e. all non-residents behave the same as residents), neither gonorrhoea nor chlamydia persists, with the median time to extinction being 4.9 years and 13.3 years for gonorrhoea and chlamydia, respectively (data not shown). Both infections can persist for up to 60 years when *A*_*s*_ is at its baseline value of 0.2, with median prevalences for gonorrhoea and chlamydia at 3.3% and 7.1%, respectively. Further increases in *A*_*s*_ result in substantial increases in the median prevalences of both infections. It should be noted that values of *A*_*s*_ higher than 0.4 are unlikely to be realistic.

Increasing *A*_*p*_ from 20% to 40% leads to higher STI prevalence at time t = 60 years (rows 2 and 5 in Figure 3), with the median gonorrhoea and chlamydia prevalences increasing from 0% to 3.6% and 6.1%, respectively. Increasing the value of *A*_*p*_ to more than 60%, however, does not result in significant changes in the prevalence of either STI.

The results from varying *A*_*d*_ suggest that gonorrhoea and chlamydia can only persist in our model if individuals remain non-resident for a period of less than 30 days. Table 5 lists the times required for elimination of gonorrhoea (first row) and chlamydia (second row) for the ranges of *A*_*d*_ shown in Figure 3; simulations for which elimination did not occur within 60 years have been omitted. This result reinforces the general observation described in the previous paragraph, whereby the time required for infection to be eliminated decreases as mobile individuals stay away from home for longer periods of time.

**Table 5:** **The impact of *A***_***d ***_**range on the time required for STI extinction, with result omitted if less than 5 extinctions occurred within 60 years over 100 simulation runs**

***A***_***d***_	**Number of eliminations (out of 100 runs)**	**Years required for elimination of gonorrhoea**	
		**Median**	**25th-75th percentile**
[7,180]	100	4.7	4.1-5.9
[14,30]	81	40.9	30.3-51.8
[14,60]	100	11.9	9.7-14.4
[14,180]	100	5.2	4.2-6.0
[30,60]	100	9.2	8.4-11.0
[30,180]	100	4.8	3.7-6.0
***A***_***d***_	**Number of eliminations (out of 100 runs)**	**Years required for elimination of chlamydia**	
		**Median**	**25th-75th percentile**
[7,180]	100	11.2	9.2-13.2
[14,60]	100	26.2	19.2-34.4
[14,180]	100	10.7	8.7-12.5
[30,60]	100	21.2	16.7-25.4
[30,180]	100	10.4	9.0-12.2

### Impact of periodic variation

**Figure 4:**
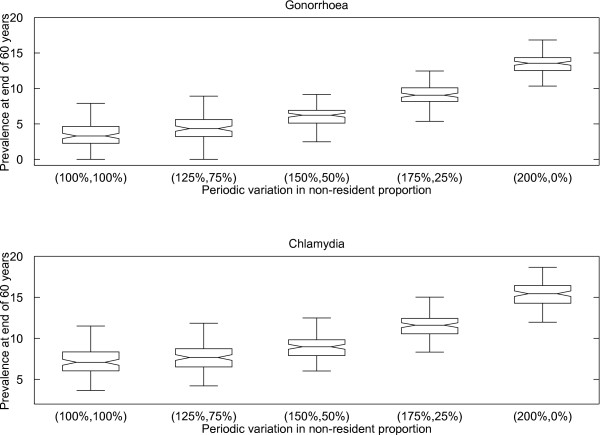
**The prevalence of gonorrhoea (top) and chlamydia (bottom) at 60 years after introduction of STI, with periodic variations in non-resident proportion.** The percentages on the horizontal axis are the percentage adjustment to the non-resident proportion for the first half and second half of the each year. For example, simulations runs in box labelled (150%, 50%), will have non-resident proportion at 1.5 times of the values listed in Table 4 during the first half of each year, and 0.5 times of the values listed during the second half of each year. See the caption of Figure 3 for the description of the box plot.

Figure 4 shows that periodic variation in mobility leads to increased prevalence. For example, prevalences of 6.4% and 8.8% are obtained for gonorrhoea and chlamydia, respectively, when the proportion of individuals away from home varies from a 50% increase to a 50% decrease over a half-yearly cycle, in comparison to 3.3% and 7.1%, respectively, when there is no change over time. If we assume population mobility follows a strict half-yearly cycle (i.e. the proportion of the population that is non-resident is twice the yearly average in first half of the year, and zero in the second half of the year), then the median STI prevalence is more than double the prevalence obtained when population mobility remains constant throughout the year.

### Impact of symptomatic treatment and return treatment

**Figure 5:**
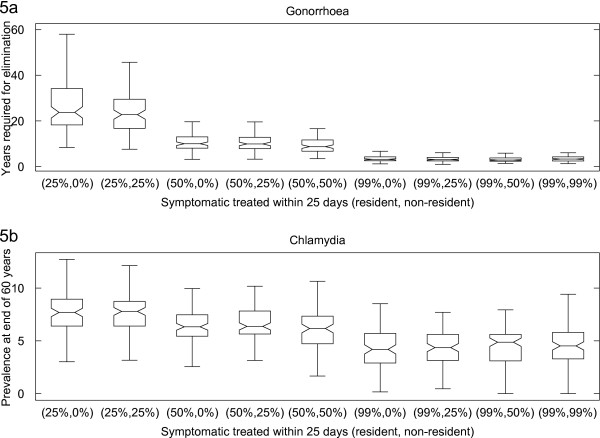
**The impact of symptomatic treatment on time required for gonorrhoea to be eliminated (Figure**5**a) and chlamydia prevalence (Figure**5**b).** The percentages on the horizontal axis are the symptomatic treatment coverage (defined as percentage of symptomatic treated within 25 days) for resident and non-resident. For example, simulation runs in box labelled (50%, 25%) had symptomatic treatment coverage of 50% for residents and 25% for non-residents. Figure 5a: number of years required for gonorrhoea to be eliminated. Figure 5b: the chlamydia prevalence 60 years since introduction of infection. See the caption of Figure 3 for the description of the box plot.

Figure 5a illustrates that symptomatic treatment is effective for controlling gonorrhoea. Gonorrhoea infection is eliminated in all but two simulation runs when 99% of symptomatic infections in residents are treated. Reducing symptomatic treatment coverage in non-residents, or excluding them altogether, increases the median time required for gonorrhoea elimination, but the increases are not significant (at 5% significance level) unless the difference in coverage between residents and non-residents is greater than 50%.

In contrast, and as can be seen from Figure 5b, symptomatic treatment was found to be ineffective in eliminating chlamydia, with chlamydia persisting at t = 60 years in more than 40% of simulation runs, even at 99% coverage for the entire population (i.e., including both residents and non-residents). Reducing symptomatic treatment for non-residents, or excluding them altogether, results in a small increase in median prevalence but the increases are not significant.

Treating people upon their return to their home location results in substantial reductions in the time required for gonorrhoea and chlamydia elimination as can be seen from Table 6. Here, a hypothetical returning treatment scheme is implemented, whereby all returning symptomatic individuals returning home and 10% of asymptomatic individuals returning home are treated. The median time for gonorrhoea elimination under this scheme is 10.3 years, compared to 17.6 years when there is no treatment on return. This strategy also results in the likelihood of chlamydia being eliminated within 60 years increasing from 16% to 27% (of 100 simulation runs).

**Table 6:** The impact of return treatment on time required for gonorrhoea and chlamydia to be eliminated and on chlamydia prevalence

**Return treatment probability (%)**	**Number of eliminations (out of 100 runs)**	**Years required for elimination of gonorrhoea**	
		**Median**	**25th-75th percentile**
0	94	25.0	17.0-36.9
5	100	10.3	7.8-13.0
10	100	10.3	7.9-14.0
**Return treatment probability (%)**	**Number of eliminations (out of 100 runs)**	**Chlamydia prevalence at the end of 60 years (%)**	
		**Median**	**25th-75th percentile**
0	0	7.0	6.0-8.7
5	2	3.5	2.1-4.6
10	27	1.0	0-2.2

For all simulation runs in the figure, residents with symptoms will be treated at a rate such that 25% of infected individuals receive treatment in 25 days (i.e. at a rate of 0.011 per day). Under return treatment, all symptomatic and a proportion (5% and 10%) of asymptomatic infected non-residents are treated upon return to their home location.

## Discussion

In our model, most gonorrhoea and chlamydia infections resolve within one year even without treatment. Furthermore, the average length of a regular partnership is two years, and only a small number of individuals acquire concurrent partners once they are in a regular partnership. Under these conditions, our results suggest that without population movement, sustained gonorrhoea and chlamydia epidemics are unlikely to occur in small populations. The addition of short-term mobility between small populations, and allowing non-residents to seek partners — despite existing partnerships in their home location— allows these STIs to persist at endemic levels.

Census data generally provide only summary information on the number of individuals and their locations at a specific point in time and do not inform us about the patterns of mobility. As demonstrated in Figure 2, different assumptions regarding population movement and sexual partner acquisition lead to different outcomes with regard to the persistence of gonorrhoea and chlamydia, even though the relative resident and non-resident proportions of the population remain largely the same. By characterising mobility and mobility related sexual behaviour with three parameters (*A*_*s,*_*A*_*p*_*and A*_*d*_*,* see Table 3), we were able to explore the theoretical impact of mobility on STI prevalence.

Allowing some non-residents, who have existing partnerships in their home location, to form new (concurrent but not co-local) partnerships when they travel, can lead to statistically significant (at 5% significance level) increases in the prevalence of gonorrhoea and chlamydia and in the probability that these STIs will persist. Partnership concurrency is known to be an important factor in STI transmission [39], and while its effect will be diminished when the overlap occurs in different geographic locations, it can still be expected to contribute to increased transmission and reinfection of regular partners. Our results suggest that the level of sexual activity away from home is a key parameter in determining whether STIs can persist in small populations. For gonorrhoea and chlamydia to persist in our model, at the levels reported for remote communities in Australia, more than 20% of individuals with regular partners are required to have partnerships away from home. Well-conducted public health strategies aimed at educating the population about safe sex when travelling have the potential to significantly reduce the prevalence of STIs in these communities or possibly lead to their elimination.

We found that gonorrhoea and chlamydia could not persist if the proportion of the population who will ever travel is below 40%, while higher proportions have only a minor additional impact on STI prevalence. This is because the model fixes the proportion of the population that is non-resident such that the number of individuals moving at any time is limited even if the percentage that does move is high. For remote Indigenous communities, if we assume the 10-12% of the population away from home during the census period [7] is representative of the entire year, then our results suggest that STIs can persist if more than 40% of individuals in the population ever travel.

In our model, gonorrhoea and chlamydia can only persist if individuals spend, on average, a fairly short time (less than 21 days for gonorrhoea and less than 30 days for chlamydia) away from home. As has been assumed in other STI models [12,21,28], we assume both gonorrhoea and chlamydia are highly infectious and partnerships of short duration are therefore sufficient to transmit infection to susceptible partners. Furthermore, if mobile individuals remain at one location for a long period, they are more likely to recover or receive treatment by the time they move again, thereby reducing the likelihood of transmitting infection to new partners at a new location or to their regular partner when returning home.

Periodic variation in mobility during the year leads to higher gonorrhoea and chlamydia prevalences. This is because the increases in prevalence during the periods of high mobility exceed the decreases that occur during low mobility periods. This result highlights the importance of accounting for periodicity in population mobility. Failing to account for this could lead to STI prevalence being underestimated and the potential impact of interventions under evaluation being overestimated. Our results indicate that this is especially important if the difference in the size of the mobile population between the high and low mobility periods is greater than 50% of the annual average.

Symptomatic treatment is more effective in eliminating gonorrhoea than chlamydia because the proportion of infections that are symptomatic is considerably higher for gonorrhoea. The expansion of treatment to non-residents, however, is predicted to provide only a small benefit under our model assumptions. This is because non-residents comprise only 10-12% of the population at any time, and only a proportion of this group will be infected and has symptoms. Furthermore, individuals are only non-resident for a short time and most of them will therefore miss out on treatment until they have returned home.

In contrast, treating all symptomatic non-residents and 10% of asymptomatic non-residents upon return to their home locations can lead to elimination of both gonorrhoea and chlamydia. A program that provides treatment to returning individuals has the advantage of encouraging a clear timeframe for individuals to seek testing and treatment but may be difficult to implement. However, if patterns of periodic mobility are known and predictable, then implementation of such a strategy could be achieved through, for example, promoting STI screening for travellers at the relevant locations and times of the year.

This study used a mathematical model to explore the potential for short-term population movement to sustain gonorrhoea and chlamydia infection at endemic levels across multiple small communities. A number of simplifying assumptions were necessary for mathematical tractability and due to gaps in available data. Firstly, in our model, individuals “select” their destinations in a probabilistic manner based on the population size of the target locations. This is based on the assumption that, in general, individuals are likely to be drawn to larger populations with more extensive facilities, opportunities for work, recreation activities, and family connections [9]. While we consider this a reasonable assumption, the extent to which this represents reality is difficult to assess due to a lack of data on mobility patterns and factors that motivate mobility. For simplicity, we also assume that all non-residents return to their home locations immediately after travel. In reality this is not necessarily the case and there is documented evidence of circular mobility [40].

We assume that the level of sexual activity is uniform across age-groups. While this may be unrealistic, we considered this to be a reasonable approach given the absence of age-specific sexual behaviour data for remote Indigenous communities of Australia. Data are especially scarce for those aged 30 years or more who make up a significantly smaller proportion of the Indigenous population [32] and are often under sampled in surveys. We have not fully investigated the effect of reducing sexual activity for older individuals in the population with our model, but preliminary simulation results (data not shown) suggest that for gonorrhoea and chlamydia to be sustainable at currently observed levels (while keeping other parameters such as number of sexual partners, size of sexually active population, and infection transmissibility within known values or realistic limits), the older age-group must be included in the sexual network. This is especially true for gonorrhoea, which is difficult to sustain in the model at endemic levels, yet an endemic prevalence of more than 10% is observed in these communities.

In the accompanying Additional file 1, under the section entitled “Age-specific sexual behaviour”, we have included a scenario whereby individuals older than 30 years no longer seek new sexual partners, but new infection is constantly introduced into the population. These simulations show that the relationships between the three mobility parameters and STI prevalence discussed here are maintained.

Finally, in order for both gonorrhoea and chlamydia to persist at realistic levels — under the same assumptions regarding the level of mobility and sexual behaviour — the average duration of chlamydia infection was required to be around 200 days. Most transmission models, however, assume this duration is greater than 300 days [13,19,41]. Our shorter duration of infection may be justified for remote communities in Australia as many of them already participate in intense STI control programs such as annual community screening [4].

## Conclusion

This study highlights the possible importance of mobility in sustaining the high prevalences of gonorrhoea and chlamydia observed in remote Indigenous communities of Australia. The proportion of short-terms visitor in the community, their duration of stay, their partner seeking behaviour and the periodicity in mobility may all contribute to the ability of these STIs to persist at a high level. Control programs that take account of these factors, such as discouraging unprotected sexual contact with visitors and encouraging visitors to seek treatment upon their return from travel, will be crucial in reducing STI prevalence in highly mobile communities.

## Competing interests

The authors declare that they have no competing interests.

## Authors’ contributions

BH participated in the design of the study, implementation and analysis of model, and drafted the manuscript. RTG and DR participated in the design of the study and drafting of the manuscript. JW provided data and information related to remote Indigenous communities and helped to draft the manuscript. DPW and DP participated in the design of the study and helped to draft the manuscript. AS, ML and JH assisted with drafting of the manuscript. All authors read and approved the final manuscript.

## Pre-publication history

The pre-publication history for this paper can be accessed here:

http://www.biomedcentral.com/1471-2334/13/188/prepub

## Supplementary Material

Additional file 1 Model overview. Click here for file
