# Supplementary material for: Population movement can sustain STI prevalence in remote Australian indigenous communities
Source: BMC Infect Dis. 2013 Apr 25;13:188. doi: 10.1186/1471-2334-13-188 (PMC3641953; doi:10.1186/1471-2334-13-188)
Supplement: Additional file 1 — Model overview. [file 1471-2334-13-188-S1.docx]

# Additional file 1

## Model overview

The modelled population consists of 5000 heterosexual individuals spanning 5 hypothetical locations. The first location is constitutes the home location for 4000 individuals, while the remaining 4 locations are home to 250 individuals each. Individuals can move to other locations throughout the simulation, but their home location does not change once it has been set.

The location, infection and partnership status of all individuals in the model is tracked daily over the course of 60 years. For simplicity, in this document as well as in the main text a month refers to a period of 30 days, and a year is a period of 12 months or 360 days.

Initially, all individuals in the model are assigned an age between 15 to 45 years. We assume all age-groups contain the same number of individuals and equal numbers of males and females. When an individual reaches the age of 45, they are removed from the population and replaced by a new individual aged 15. The new individual is assigned the same gender, home location, and sexual behaviour (see next section) as the individual who has been removed. New individuals enter the model at their home location. A removed individual does not seek new partners, but can still engage in sex with existing partners until the partnerships expire.

Each simulation run begins with a burn-in period of 10800 days, followed by introduction of gonorrhoea and chlamydia infection into the population. The number of infections introduced is based on data from the STRIVE baseline prevalence study [1], which is summarised in Table A-1 below.

| Gender | STI | Prevalence by Age (in years) | | | |
| --- | --- | --- | --- | --- | --- |
|  |  | 15 - 19 | 20 - 24 | 25 – 29 | 30 – 34 |
| Male | Gonorrhoea | 13.7 | 6.5 | 4.0 | 4.1 |
|  | Chlamydia | 11.8 | 10.4 | 7.4 | 4.6 |
| Female | Gonorrhoea | 13.5 | 7.6 | 2.8 | 4.3 |
|  | Chlamydia | 17.4 | 8.2 | 6.0 | 3.5 |

Table A‑1: Initial STI prevalence

In the model the initial prevalence for the older age group (35+) is assume to be 0 for both infection, although this cannot be maintained due to aging of the infected individuals.

After the introduction of infection, simulations are run for 21600 days (or 60 years), and the number of individuals infected at the end of each simulation, as well as the time to extinction of infection (if this occurs) are recorded.

Note that the prevalence described above does not correspond to the equilibrium prevalence of the model. Even in the baseline case, where population-wide prevalence remains largely stable over the course of 60 years, STI prevalences are overestimated in the older age-groups and underestimated in the younger age-groups. More specifically, because most published sexual behaviour studies for Indigenous populations in Australia (on which this model is based) are not stratified by age and do not provide on the age of partners of study participants. It was therefore not possible to accurately capture age-specific sexual behaviour such as changes in the level of sexual activity and assortativity in the selection of sexual partners by age. Given the aim of this study is to focus on the possible role of mobility in sustaining a high endemic levels of infection in hypothetical remote communities, we opted for a rather simplistic implementation of sexual behaviour in the model. In accordance with existing practice in many remote Indigenous communities in Australia, 44% of the population is screened and treated annually for gonorrhoea and chlamydia [2]. Individuals are only be screened when they are at their home location, and no individual is screened more than once per year.

## Formation of partnership and sexual behaviour

At each time step sexual partnerships can form between individuals who are currently in the same location. Sexual acts can take place and transmissions occur within partnerships only when both partners are in the same location at the same time. Partnerships are maintained even if the partners move to different locations and sexual activity can resume when they are again located in the same location.

The frequency at which new partners are sought, and the length and type of partnership (regular or casual) formed for each individual are assigned on the basis of results from Bryant *et al*. [3]. In the model, 36.5% of the population can form regular partnerships only. Of the remaining 63.5% of the population, 27.6% can form concurrent partnerships, and can seek a casual partner even if they are already in a regular partnership.

Individuals that can form concurrent partnerships can have only one regular partner and one casual partner at the same time, while the remainder can have one regular or casual (if allowed) partner at the same time. The length of regular partnerships is determined by a random draw from an exponential distribution with average of 2 years. Casual partnerships are terminated when one of the partners seeks new partners (see below).

Individuals seek partners at different rates. From the results of Bryant *et al*. [3], we assume that 60% of the population only seeks one partner per 6 months. The remaining 40% of the population seek more than one partner per 6 months. For individuals who seek more than one partner in six months, the number of partners sought is drawn from a Poisson distribution with mean of 8. Data are not available to inform the choice of this distribution and parameter (the survey only reported the proportion of the population having more than one partner in 6 months), however changing the distribution means does not lead to statistically significant changes in median STI prevalences unless it is reduced to 2 or less (see Figure A-1 below). This is because even if an individual seeks partners frequently, quite often they are unable to find a suitable partner due to the relatively small population size.

Figure A‑1: STI prevalence in the model versus the average number of partners per six months (for individuals who seek more than 1 partner in six months).

Individuals seek partners based on a 6-month (= 180 days) window. For example, if an individual can seek $n$ partners in 6 months, then they will and seek partners (and form a partnership if a suitable partner is found) on the $a_{1}, a_{2}\ldots a_{n}$-th days within the 180 days window, with $a_{1}, a_{2}\ldots a_{n}$ drawn from a uniform distribution from 1 to 180. This process is repeated as long as the individual remains in the modelled population, with a new set of $a_{1}, a_{2}\ldots a_{n}$ generated every 180 days.

An individual scheduled to seek a new partner if they are currently single (i.e. not in any partnership), only has a regular partner but they can form concurrent partnership, or under influence of parameter$A_{s}$ (see main text). If an individual is scheduled to seek a new partner, then their existing casual partnership is terminated, but their former casual partner will remain single until they themselves are scheduled to seek a new partner. For individuals who only seek one partner per 6 months, the average length of a casual partnership will be 6 months, although in the actual simulation runs the duration of casual partnerships can range from 1 day to 12 months. Regular partnerships are not affected by the partner seeking schedule: an individual who is already in a regular partnership will not seek a new partner unless they are able to form concurrent partnerships.

Partnerships can only form between individuals if both are seeking partners at the same location at the same time. The type of partnership formed is determined by the preferences of the individuals involved. For example, let us assume individual $A$ can form regular partnerships only, individual $B$ can form casual or regular partnerships, and individual $C$ can form concurrent partnerships, and all three individuals are seeking partners at the same location as the same time (for this example, we will ignore age and gender). In this case, any partnership involving individual $A$ must be regular; individual $B$ can form regular or casual partnerships as long as they don’t have a regular partner already (and any casual partnership will have been terminated since individual$B$ is seeking a new partner); and if individual $C$ already has a regular partner, then they can only form a casual partnership with individual$B$. For cases where either regular or casual partnerships are possible (e.g. both individual $B$ and individual$C$ have no regular partners), the probability of forming a regular partnership rather than a casual partnership is assumed to be 0.25 in the baseline model, although a sensitivity analysis showed that the impact of this assumption is not significant over the course of 60 years (data not shown).

If, during the search for a new partner, more than one potential partner is available, priority is given to the partnership where the age differences between male and female partners is closest to 3 years, based on findings reported in a study of sexual behaviour in Indigenous populations by Smith et al [4]. However, given the small population size, the choices of possible partners are often limited in the model and the age difference of 3 years cannot always be maintained.

Sexual acts can only occur within a partnership if both partners are in the same location at the same time. The frequency of sex is 0.429 per day or around 3 acts per week [5]. Condoms might be used during sex, and the efficacy of condoms in preventing STI transmission is assumed to be 100%. Condom usage is based on data reported in the study by Bryant *et al*. [3] (see below).

In this model, we assumed that the presence of symptoms can influence condom usage. In the absence of symptoms, the condom usage rate was calculated based on data [3] for the proportion of individuals that “always” use condoms, 0.206 and 0.392, for regular and casual partnerships respectively. If either partner has symptoms, condom usage was calculated as one minus the proportion that “never” used condom, i.e., 0.567 and 0.801, for regular and casual partnerships, respectively..

### Age-specific sexual behaviour

One of the difficulties in modelling STI transmission in remote Indigenous communities of Australia is the lack of data on sexual behaviour, particularly for the older age-group. This lack of data is due to the fact that the majority of studies of sexual behaviour study in these communities have focused on the younger population aged 15 to 30. At the other end of the age spectrum, the STI prevalences of remote Indigenous population are consistently very high (more than 10%) within the 15-19 age-group, and close to double the prevalences observed for the 20-24 age-group [1]. We found it difficult to replicate and maintain this prevalence profile long-term in the modelled population without drastically changing of the values of natural history parameters outside the ranges supported by the literature or substantially lowering the age of sexual debut. In the absence of data to support such assumptions these changes are not considered for this model.

The main focus of this study is on the role of mobility in sustaining STI prevalence in small remote populations. Given the lack of age-specific data on the sexual behaviour of remote Indigenous populations, we have made significant assumptions regarding sexual behaviour in the model. We assumed sexual behaviour doesn’t change for individuals as they aged beyond 30. This is arguably an unrealistic but simulation results suggest that it is necessary in order to generate robust results for small populations, as infection often becomes extinct (due to the lack of susceptible individuals in the population) if the older age-groups are excluded.

As a result of this assumption, the age-specific sexual behaviour (and as a consequence, the age-specific STI prevalence) of the older age-groups is likely to be overestimated in the model. For example, the top row of Figure A-2 shows the age-specific partnership frequency distribution and prevalence of the baseline model used in the main text. While the STI prevalences are fairly similar to levels shown in Table A-1 if all 15-30 years-olds are grouped as one age-group, further division has shown that the model has underestimated the prevalences of those age less than 20, and overestimated the prevalences for those older than 20.

In order to address this issue, another set of results have been generated with the following assumptions:

1. Individuals will not seek any more new sexual partners once they are aged 30 or older. This can be considered complementary to the results in the main text where sexual behaviour doesn’t change for individual aged over 30.
2. Some individuals, based on the data shown in Table A-1, are already infected with STI when they enter the population at age 15. This is necessary as preliminary simulation results (not shown) suggest that infection cannot be sustained at observed prevalence levels (especially for gonorrhoea) when a large proportion of the infected population is ‘lost’ to the modelled population due to aging. Note that under this assumption, the extinction of infection, one of the benchmarks used in the main text for describing the impact of mobility, never occurs.

As shown in the bottom row of Figure A-2, with these two assumptions in place, the number of partners for individuals aged 30+ is reduced and there is consequently a reduction in the age-specific STI prevalences for the older age-group. Note that the number of partners and the age-specific STI prevalences are still relatively uniform among the 15-30 age-groups, which still does not fully correspond to the results shown in Table A-1. Therefore it is highly likely that the sexual behaviour is not uniform even within the 15-30 age-group, but further adjustment could not be made without making additional assumptions in the absence of detailed sexual behaviour data.

Figure A-3 shows the prevalence of gonorrhoea and chlamydia at 60 years (averaged over 100 simulations runs) if the two assumptions described above are enforced in the model, with parameter values equal to the baseline parameter value used in the main text (i.e.$A_{s}=0.2,A_{p}=100\%, A_{d}=[14,21]$). The results shown in Figure A-3 are mostly consistent with the results and conclusions drawn from Figure 3 of the main text, with the only exceptions being the number of extinctions (never occur due to the second assumption), and the lower population-wide prevalence, due to the inclusion of less sexually active older age-groups.


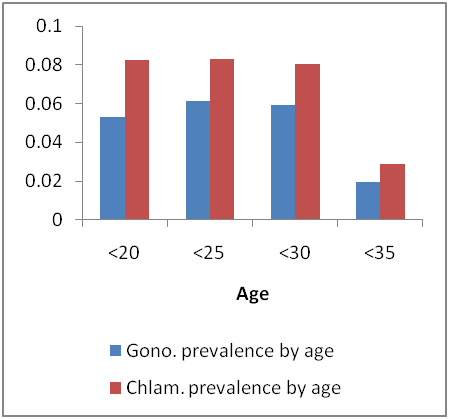

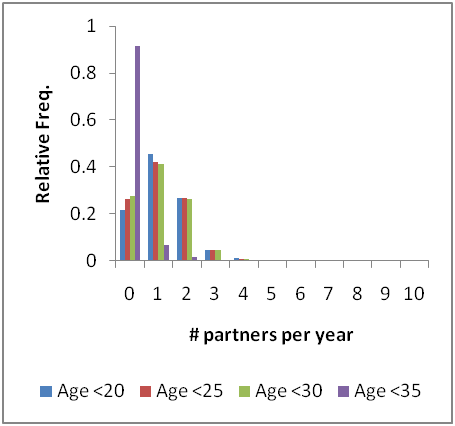

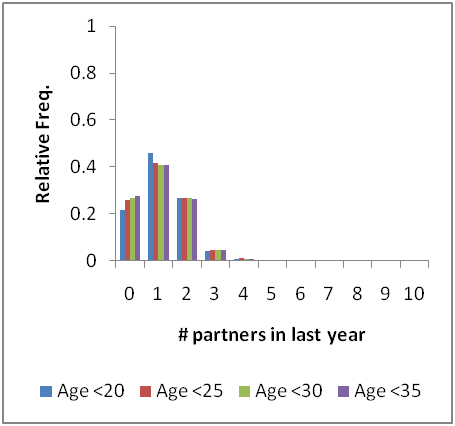

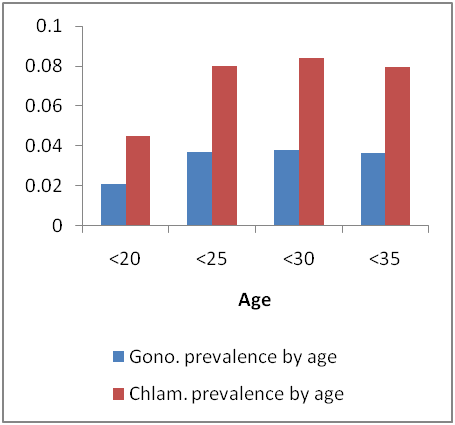


Figure A‑2: Number of partners per year and prevalence by age for selected age-groups with parameter values of$\boldsymbol{A}_{\boldsymbol{s}}\boldsymbol{=0.2,}\boldsymbol{A}_{\boldsymbol{p}}\boldsymbol{=100\%,}\boldsymbol{A}_{\boldsymbol{d}}\boldsymbol{=[14,21]}$, without (top row) and with (bottom row) the enforcement of the two assumptions (i.e. individuals will not seek any more new sexual partners once they are aged 30 or older and non-zero prevalence at age of 15, see text)

Figure A‑3: The prevalence of gonorrhoea and chlamydia at 60 years under different $\boldsymbol{A}_{\boldsymbol{s}}$ (first and fourth rows), $\boldsymbol{A}_{\boldsymbol{p}}$ (second and fifth row) and $\boldsymbol{A}_{\boldsymbol{d}}$ range (third and sixth row), with the two assumptions enforced (i.e. individuals will not seek any more new sexual partners once they are aged 30 or older and non-zero prevalence at age of 15, see text). See Figure 3 of the main text for definition of boxes, notches and whiskers.

## Natural history of gonorrhoea and chlamydia

During sexual partnerships with infected partners, individuals can become infected with gonorrhoea or chlamydia. Transmission occurs with a probability determined by the number of sex acts that take place and the per-act probability of transmission. The progression and resolution of infection is tracked over time.

The transmissibility and natural history of gonorrhoea and chlamydia (including gender-specific durations of infection and immunity, and per-act transmission probability) have not been fully elucidated and a wide range of values for natural history parameters are quoted in the published literature [6]. For this model we assumed that the acquisition and progression of infection for both gonorrhoea and chlamydia followed identical pathways and the two infections were differentiated by the appropriate choice of parameter values (e.g., transmission probability, duration of infection, proportion of infections that are symptomatic). Individuals enter the sexually active population susceptible to infection (i.e., uninfected)^[[1]](#footnote-2)^. Susceptible individuals can acquire infection through sexual contact whereupon they initially enter the ‘Exposed’ state and are not yet infectious. Following this period of latency, exposed individuals then become ‘Infectious’ either asymptomatically or symptomatically and, in the latter case, may receive treatment and return to the susceptible state.

We assume that screening sensitivity and treatment efficacy are 100% for both chlamydia and gonorrhoea. We consider this to be a reasonable simplifying assumption based on the diagnostic techniques and treatments currently available for remote communities of Australia [7-10]. In the absence of treatment, an Infectious individual eventually recovers naturally to an immune state in which they are neither infectious nor susceptible to infection. Following the loss of immunity, individuals return to the susceptible state.

We assume the mean duration for untreated asymptomatic and symptomatic gonorrhoea infections are the same for both STIs, as was assumed in the models developed by Johnson et al [6]. While some STI models (e.g., gonorrhoea, Garnett *et al.* [11]) assumed that the duration of symptomatic infection is shorter than asymptomatic infection, this approach is not taken in our model. This is because the basis for assuming a shorter duration is the assumption that those with symptomatic infection can and will seek treatment s**.** Given that access to healthcare is known to be limited in remote Indigenous communities of Australia [12], and that changes in the proportion of symptomatic infections for which it is assumed treatment is sought is one of the parameters examined in this model, we have assumed that the duration of gonorrhoea infection is the same for asymptomatic and symptomatic infection, and set symptomatic treatment to zero for the baseline case.

## Individual movement

This study focuses on temporary mobility between multiple small populations. Only movements of short duration are modelled. All initial mobility entails the movement of individuals away from their home location to a new location. Individuals stay at the new location for a pre-determined period before returning to their home location. Cyclic mobility (moving from one non-home location to another), while known to occur in some remote Indigenous communities [13], is not considered in this study due to a lack of data.

At each time step, the number of individuals to move is determined by the proportion of the population in each location that is non-resident based on the findings reported in the study by Biddle and Prout [14]. For example, let us assume that at time step *t*, there are 500 males aged 15 to 20 years within the population. Based on Table 3 of the main text, 50 of them should be away from their home at any given time. Let us also assume that there are 45 males aged 15 to 20 years actually away from their home location at this time. This means that at the next time step (i.e. *t*+1), 5 males aged 15 to 20 will move away, bringing the number to 50 at time step *t*+1. During a simulation, the exact number of non-residents (and hence number of movements ) can vary at each time step, due to non-residents returning home, the aging of the population, as well as periodic variations in mobility (when modelled).

We assume that an individual moving away from home will select their new destination based on a random selection process. This selection will be weighted by the population size (including both number of residents and non-resident) of each location. For example, an individual will be twice as likely to move to a location with 500 individuals as to one with 250 individuals. This is a simplification of the gravitational formula commonly used in modelling mobility [15].

### Relationship between individual movements and the three parameters ($\boldsymbol{A}_{\boldsymbol{s}}\mathbf{,}\boldsymbol{A}_{\boldsymbol{p}}\mathbf{and}\boldsymbol{A}_{\boldsymbol{d}}$)

The three parameters$A_{s},A_{p}\mathrm{and}A_{d}$ discussed in the main text describe the level of individual mobility at the population level. In this section we will examine how these three parameters are related to movement at the individual level. This is summarised in histograms shown in Figure A-4. This figure shows the relative number of individuals that have a certain number of trips and away partners (defined as partners an individual has while away from home) within one year.

The top row of Figure A-4 examines the effect of$A_{s}$ on individual mobility. Since $A_{s}$does not influence an individual’s movement in the model (it only governs how an individual behaves when they are away from home), it is not surprising that$A_{s}$ does not influence the number of trips. However, as expected, it does influence how many partners an individual has while away from home. Note that it is still possible for individuals to have away partners even if$A_{s}=0$, as they are free to form partnerships in any location as long as they do not already have a partner.

The middle row of Figure A-4 shows how individual mobility changes with different values of$A_{p}$. In terms of the number of away trips, a decrease in $A_{p}$ leads to an increase in the number of individuals who have more trips per year. This is due to a condition set in the model that ensures that the number of individuals away from home at anytime is constant. If $A_{p}$ is small, only a small number of individuals are allowed to move. Since the duration of time they stay away from home is fixed (by$A_{d}$), the only way to maintain the previous condition is for them to move more often, hence more frequent travel (and a the shift to the right) as $A_{p}$ decreases. In terms of the number of away partners, a reduced $A_{p}$ does lead to more individuals having more away partners. This is due to the fact that potential away partners have to be engaged with a smaller number of mobile individuals.

The impact of adjusting$A_{d}$ on individual mobility is shown in the bottom row of Figure A-4. Similarly to the reasoning above, a longer $A_{d}$ means individuals have to move less frequently in order to maintain the number of individuals away from home at a constant level. This leads to a decrease in the number of away trips per year (and a shifting of the histogram to the left) as$A_{d}$ increases. If$A_{d}$ is very high (say, a year or more), then it is quite likely that only a small number of movements can occur within a year, and the population can be considered as non-mobile. The effects of changing$A_{d}$ on the number of away partners is limited because any increase in the number of away partners due to increased away duration is compensated for by the decrease in the number of individuals that can move in a year.


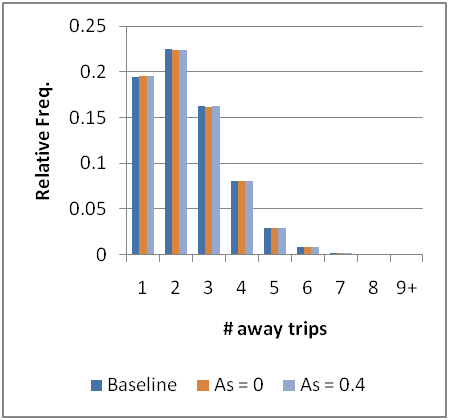

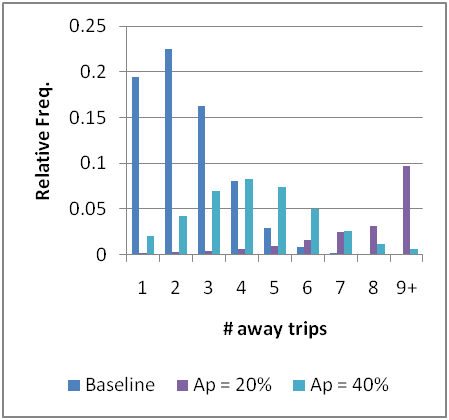

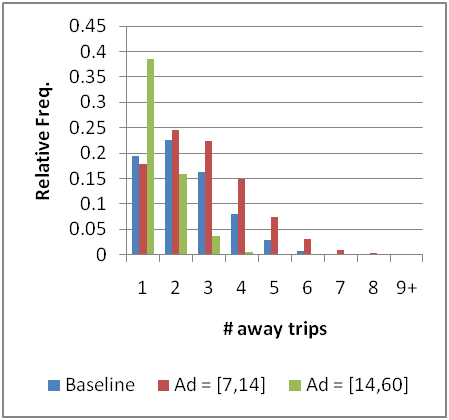

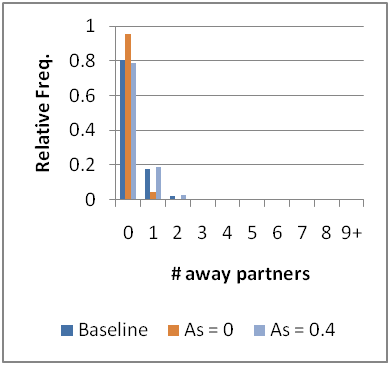

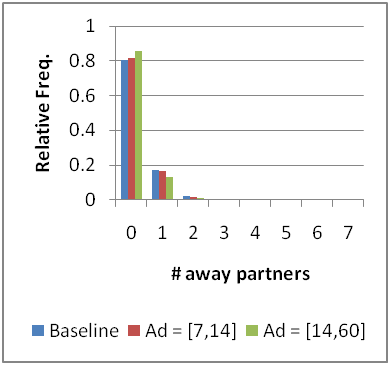

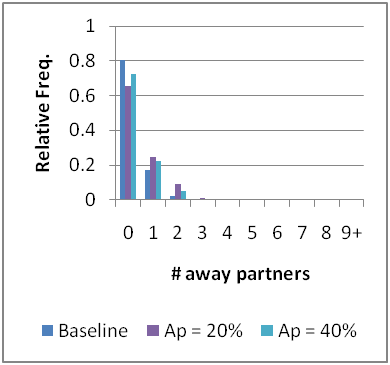


Figure A‑4: The relative number of individuals (over 100 simulation runs) who have a certain number of away trips (left column) and the number of away partners (right column) in one year under selected scenarios. For the baseline case, $\boldsymbol{A}_{\boldsymbol{s}}\boldsymbol{=0.2,}\boldsymbol{A}_{\boldsymbol{p}}\boldsymbol{=100\%,}\boldsymbol{A}_{\boldsymbol{d}}\boldsymbol{=[14,21]}$

## Spread of infections across locations

The analysis in the main text only focuses on population-wide prevalence. However, from a healthcare point of view, one of the results of interest is how infections are distributed across locations. For example, if infection is localised to a particular location, then it might be more efficient to focus interventions for control and prevention at these locations rather than across the entire population.

Due to a lack of detailed data on mobility patterns for residents of remote Indigneous populations in Australia, we have made a number of simplifying assumptions. A detailed analysis of location-based interventions might is therefore premature until additional data become available. However, under the existing assumptions, some observations can be made and some insights gained.

Figure A-5 shows how individuals and infection are distributed across locations at the end of our simulation (averaged over 100 simulation runs). In the top row, the three parameters are at their baseline values ($A_{s}=0.2,A_{p}=100\%, A_{d}=[14,21]$), and the results show that while 74% of the population is at Location 0 (L0), more than 80% of the infections are located at L0. This suggests that under the baseline setting, a disproportionate level the infection is concentrated at the biggest location, and hence it might be more efficient to allocate more screening and treatment resources to that location. Similar analysis on other simulation runs (not shown) show that this pattern does not change substantially as long as $A_{p}$ remains at the baseline value of 100%. In the second row of Figure A-5,$A_{p}$ was reduced to 40%, and the proportion of infection at L0 is reduced to 76%. Additional simulations show that the proportion infected at L0 can be reduced even further if$A_{p}$ is reduced further (for example, for$A_{p}=20\%$, only 69% of chlamydia infection is at L0, data not shown), however these results may be skewed due to a lower level of infection in general once $A_{p}$ is reduced below a certain level (for example, gonorrhoea extinction occurs at$A_{p}=20\%$)

The concentration of infection at the more populous location in our model could be due to a combination of two factors: 1) Mobile individuals have a wider selection of potential partners (and if$A_{s}\neq0$, they can have more partners as well) than a non-mobile individual, and therefore they are more likely to be involved in the transmission of infection; 2) Since individuals moving away from home are more likely to go to a more populous location in our model, mobile individuals are likely to gather at populous locations.

Note that one consequence of reducing $A_{p}$ is an increase in the frequency of travel (see previous section on the relationship between individual movement and$A_{p}$). However, this does not lead to an increase in overall STI prevalence (see main text) or an increase in the proportion of the population infected at L0. In contrast, another method to increase the frequency of travel is to reduce$A_{d}$, and this adjustment does lead to an increase in overall STI prevalence (see main text) and an increase in the proportion infected at L0 (around 85% of infections are at L0 if $A_{d}=[7,14]$, bottom row of Figure A-5). We suspected these different outcomes are due to differences in the number of movable individuals. In the former case, the increased travel frequency was only restricted to a smaller number of individuals, therefore transmission of infection across different locations is restricted by a smaller number of mobile individuals and their associated contacts. In the latter case, since the increase in travel frequency includes the entire population, there are more distinct mobile individuals, and infections are more likely reach a larger group of susceptible individuals across different locations.


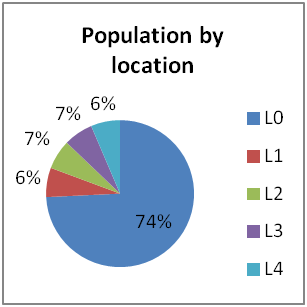

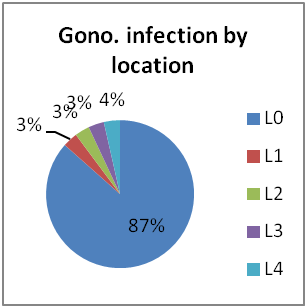

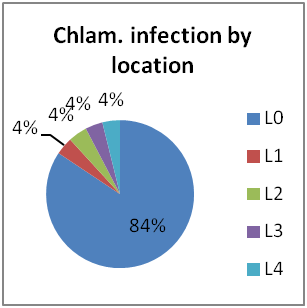

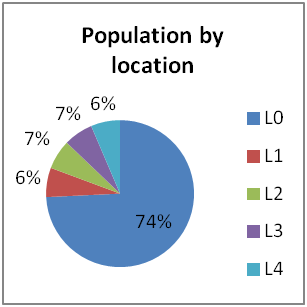

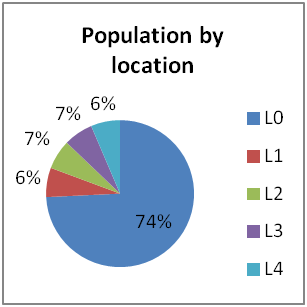

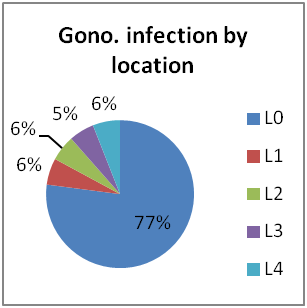

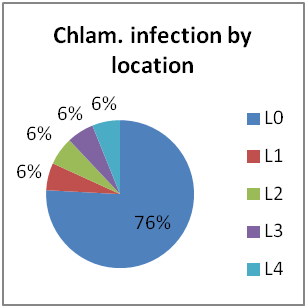

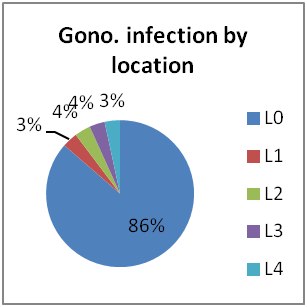

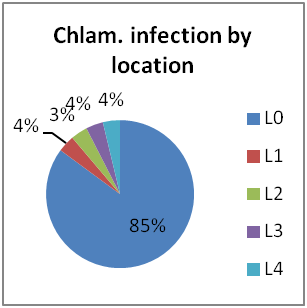


Figure A‑5: Distribution of population and STI prevalences across all location at the end of simulation (averaged over 100 runs). Top row: Baseline case ($\boldsymbol{A}_{\boldsymbol{s}}\boldsymbol{=0.2,}\boldsymbol{A}_{\boldsymbol{p}}\boldsymbol{=100\%,}\boldsymbol{A}_{\boldsymbol{d}}\boldsymbol{=[14,21]}$). Middle row:$\boldsymbol{A}_{\boldsymbol{s}}\boldsymbol{=0.2,}\boldsymbol{A}_{\boldsymbol{p}}\boldsymbol{=40\%,}\boldsymbol{A}_{\boldsymbol{d}}\boldsymbol{=[14,21]}$. Bottom row: $\boldsymbol{A}_{\boldsymbol{s}}\boldsymbol{=0.2,}\boldsymbol{A}_{\boldsymbol{p}}\boldsymbol{=40\%,}\boldsymbol{A}_{\boldsymbol{d}}\boldsymbol{=[7,14]}$

## References

1. Guy R, Garton L, Taylor-Thompson D, Silver B, Hengel B, Knox J, McGregor S, Rumbold A, Ward J, Kaldor J: **The 2010 baseline prevalence study conducted by the STRIVE trial.** In *Book The 2010 baseline prevalence study conducted by the STRIVE trial* (Editor ed.^eds.). City; 2011.

2. Guy R, Ward J, Smith K, Su J-Y, Huang R-L, Tangey A, Skov S, Rumbold A, Silver B, Donovan B, Kaldor J: **The impact of sexually transmissible infection programs in remote Aboriginal communities in Australia: a systematic review.** *Sexual Health* 2011.

3. Bryant J, Ward J, Worth H, Hull P, Solar S, Bailey S: **Safer sex and condom use: a convenience sample of Aboriginal young people in New South Wales.** *Sexual Health* 2011, **8:**378-383.

4. Smith K, Watson C, Senior K, Latif A: **Central Australian STI risk factor study.** In *Book Central Australian STI risk factor study* (Editor ed.^eds.). City: Sexual Health and BBV Unit, Centre for Disease Control, Department of Health anf Families,Northern Territory Government; 2008.

5. Rissel CE, Richters J, Grulich AE, de Visser RO, Smith AMA: **Sex in Australia: selected characteristics of regular sexual relationships.** *Australian and New Zealand Journal of Public Health* 2003, **27:**124-130.

6. Johnson LF, Alkema L, Dorrington RE: **A Bayesian approach to uncertainty analysis of sexually transmitted infection models** *Sexually Transmitted Infections* 2010, **86:**169-174.

7. Wasik M, Djuric Kachli M: **A review of common sexually transmitted diseases.** *Formulary* 2009, **44:**78-86.

8. Rockett R, Goire N, Limnios A, Turra M, Higgens G, Lambert SB, Bletchly C, Nissen MD, Sloots TP, Whiley DM: **Evaluation of the cobas 4800 CT/NG test for detecting Chlamydia trachomatis and Neisseria gonorrhoeae.** *Sexually Transmitted Infections* 2010, **86:**470-473.

9. Tabrizi S, Twin J, Unemo M, Limnios EA, Guy R: **Analytical performance of GeneXpert® CT/NG, the first real-time PCR point-of-care assay for the detection of Chlamydia trachomatis and Nesseria gonorrhoeae.** In *Book Analytical performance of GeneXpert® CT/NG, the first real-time PCR point-of-care assay for the detection of Chlamydia trachomatis and Nesseria gonorrhoeae* (Editor ed.^eds.). City; 2012.

10. Lahra M, Tapsall J: **Annual Report of the Australian Gonococcal Surveillance Programme,2009.** *Communicable Diseases Intelligence* 2010, **34:**89-95.

11. Garnett GP, Mertz KJ, Finelli L, Levine WC, St Louis ME: **The transmission dynamics of gonorrhoea: modelling the reported behaviour of infected patients from Newark, New Jersey.** *Philosophical Transactions: Biological Sciences* 1999, **354:**787-797.

12. Prout S: **The entangled relationship between Indigenous spatiality and government service delivery.** In *Book The entangled relationship between Indigenous spatiality and government service delivery* (Editor ed.^eds.). City: Centre for Aboriginal Economic Policy Research, ANU; 2008.

13. Prout S: **On the move? Indigenous temporary mobility practices in Australia.** In *Book On the move? Indigenous temporary mobility practices in Australia* (Editor ed.^eds.). City: Centre for Aboriginal Economic Policy Research, ANU; 2008.

14. Biddle N, Prout S: **The geography and demography of Indigenous temporary mobility: an analysis of the 2006 census snapshot.** *Journal of Population Research* 2009, **26:**305-326.

15. Signorino G, Pasetto R, Gatto E, Mucciardi M, La Rocca M, Mudu P: **Gravity models to classify commuting vs. resident workers: an application to the analysis of residential risk in a contaminated area.** *International Journal of Health Geographics* 2011, **10**.

1. Apart from the alternate implementation with the assumption of non-zero prevalence at age 15, as described in previous section) [↑](#footnote-ref-2)
